# Narcolepsy as an Immune-Mediated Disease

**DOI:** 10.1155/2014/792687

**Published:** 2014-01-14

**Authors:** Alberto K. De la Herrán-Arita, Fabio García-García

**Affiliations:** ^1^Stanford Center for Sleep Sciences and Medicine, Stanford University School of Medicine, 3165 Porter Drive, Palo Alto, CA 94304, USA; ^2^Instituto de Ciencias de la Salud, Departamento de Biomedicina, Universidad Veracruzana, Industrial-Animas, 91190 Xalapa, VER, Mexico; ^3^Laboratory of Sleep Biology, Department of Biomedicine, Institute of Health Sciences, Veracruzana University, Avenida Luis Castelazo Ayala s/n, Industrial-Animas, 91190 Xalapa, VER, Mexico

## Abstract

Narcolepsy is a neurological disorder characterized by excessive daytime sleepiness, cataplexy, hypnagonic hallucinations, sleep paralysis, and disturbed nocturnal sleep patterns. This disease is secondary to the specific loss of hypothalamic hypocretin (orexin)-producing neurons in the lateral hypothalamus. An autoimmune basis for the disease has long been suspected based on its strong association with the genetic marker DQB1∗06:02, and current studies greatly support this hypothesis. Narcolepsy with hypocretin deficiency is associated with human leukocyte antigen (HLA) and T cell receptor (TCR) polymorphisms, suggesting that an autoimmune process targets a peptide unique to hypocretin-producing neurons via specific HLA-peptide-TCR interactions. This concept has gained a lot of notoriety after the increase of childhood narcolepsy in 2010 following the 2009 H1N1 pandemic (pH1N1) in China and vaccination with Pandemrix, an adjuvanted H1N1 vaccine that was used in Scandinavia. The surge of narcolepsy cases subsequent to influenza A H1N1 infection and H1N1 vaccination suggests that processes such as molecular mimicry or bystander activation might be crucial for disease development.

## 1. Introduction

Narcolepsy with hypocretin deficiency is a common sleep disorder that affects approximately 0.02% of the population worldwide and causes disability in 24% of the affected subjects. It is clinically characterized by excessive daytime sleepiness and abnormal sleep-wake patterns. These patients also suffer from cataplexy, a sudden loss of muscle tone triggered by strong emotions such as laughter, and are considered to be fragments of Rapid Eye Movement (REM) sleep that intrude into wakefulness, such as hypnagogic (dream-like) hallucinations as they drift off to sleep, as well as cataplexy (sudden loss of muscle tone triggered by strong emotions). All narcoleptic subjects present chronic sleepiness, but the intensity varies across the day and between individuals. This sleepiness is most troublesome during periods of inactivity, though it is often improved temporarily by a brief nap. As a consequence of sleepiness, patients may report inattention, poor memory, blurry vision, diplopia, and automatic behaviors such as driving without awareness [1–3].

## 2. The Hypocretin System

The disorder is caused by the specific loss of hypothalamic neurons producing two hypocretin peptides with high homology with each other, namely, hypocretin-1 and hypocretin-2 (also called orexin A and B), which are comprised of 33 and 28 amino acids, respectively [4–6]. These are produced by proteolytic cleavage of a single precursor protein known as preprohypocretin. There are two cloned hypocretin receptors, HCRT1R and HCRT2R, both of which are serpentine G-protein-coupled receptors [5]. Hypocretin-secreting neurons project from the LH throughout the central nervous system (CNS) to neurons involved in the regulation of feeding, sleep-wakefulness, neuroendocrine homeostasis, and autonomic regulation [7]. Hypocretin knockout mice and dogs with null mutations in the HCRT2R gene develop narcolepsy, indicating that the loss of this peptide is causal for development of the disease [8–10].

Moreover, narcoleptic patients typically have low hypocretin cerebrospinal fluid (CSF) levels, which can be explained by the loss of over 90% of their hypocretin-producing neurons [11–14]. This loss of hypocretin-producing cells is selective rather than general or regional destruction, as intermingling-melanin concentrating hormone (MCH)-producing neurons appear to be unaffected in the same narcoleptic patients [13, 14]. This specific depletion of hypocretin-secreting neurons led to the hypothesis that narcolepsy is an autoimmune driven process within the hypothalamus.

## 3. The Immune System and Narcolepsy

An autoimmune basis for the hypocretin cell loss in narcolepsy has long been suspected based on its strong genetic association with selected HLA alleles [15]. These alleles encode multiple subtypes of Major Histocompatibility Complex (MHC) classes I and II proteins, which present foreign peptides to T cells during infections, triggering immune responses via TCR activation. In the case of autoimmunity, self-peptides are hypothesized to be mistakenly recognized as foreign, leading to tissue destruction, often occurring in context of specific HLA alleles.

Among autoimmune diseases, narcolepsy may be uniquely positioned to demonstrate autoimmunity in humans. First, narcolepsy occurs nearly exclusively with DQ0602, a heterodimeric a/b class II protein encoded by HLA DQB1∗06:02 and DQA1∗01:02, two gene variants found together on the same haplotype [15]. Second, a specific amino acid variant in the T cell receptor alpha (TCR@) locus J24 segment encodes the chain of the heterodimeric a/b TCR molecule also confer increased risk [16], indicating a crucial role for TCR containing this segment in the immunological synapse in narcolepsy. Finally, studies have shown increased rates of narcolepsy onset in children following exposure to streptococcus pyogenes [17], selected H1N1 vaccine preparations [18–20], and influenza A H1N1 infections [21]. These findings strongly suggest that some T cells that can be activated by H1N1 epitopes also lead to hypocretin neuron destruction. A parsimonious explanation would involve mimicry between H1N1 and hypocretin peptide sequences, as hypocretin is the only known protein specific for these cells.

Clinically, the association between infections and autoimmune disorders is a well-known phenomenon. Viral, bacterial, and parasitic pathogens may cause autoimmune inflammation of a variety of organs, including the heart and bowel, as well as the peripheral nervous system and the CNS.

## 4. Autoimmunity and Narcolepsy

Characterization of the cellular and molecular basis of hypocretin cell death in narcolepsy is extremely important both to understanding the pathogenesis and to achieving the ultimate goal of designing specific treatments. Although immune responses are complex, involving both humoral and cellular immune components, some autoimmune diseases are predominately CD4^+^ T cell mediated, whereas others seem to be primarily antibody mediated.

Viruses and other infectious insults are implicated in the etiology of many human autoimmune diseases. In the case of narcolepsy, streptococcus pyogenes and influenza A H1N1 infection and H1N1 vaccination have been strongly correlated to the onset of the disease [17–21]. There are various mechanisms by which infection can lead to the initiation of an autoimmune response; however, intense efforts to identify an underlying pathogen have failed in the vast majority of autoimmune disorders. Two major hypotheses have been raised to explain how infections may induce autoimmunity in the CNS and more specifically hypocretin-neuron destruction in narcolepsy: bystander activation and molecular mimicry. Although infectious diseases may trigger cerebral autoimmune diseases by other mechanisms, such as activation of CNS antigen-presenting cells (APCs) or inhibition of immunosuppressive cytokines, bystander activation and molecular mimicry are currently the most intriguing and most likely mechanisms.

## 5. Bystander Activation of Autoreactive T Cells

It is well known that cytotoxic T cells are polyclonally stimulated during viral infections. Cytokines secreted by antigen-responsive cells at infectious foci may directly stimulate surrounding T cells by cytokines in the absence of direct triggering of the T cell receptor [22]. Thus, a proinflammatory microenvironment creates a fertile field, allowing activation of, but not pathogen-specific T cells, which subsequently may damage hypocretin cells [23]. This latter scenario may become particularly relevant when underlying infection causes tissue destruction, thereby deliberating host cell proteins, which can be presented by APCs to autoreactive bystander T cells.

In addition, epitope spreading may induce autoreactive hypocretin T cells. During infection, pathogen-specific T cells develop in a hierarchical manner, being directed against immunodominant epitopes first. Subsequently, the T cell response may be generated against further, less dominant epitopes of the same protein or against epitopes of a different protein such as a hypocretin cell autoantigen. Such epitope spreading is useful for the host to optimize a T cell response during an ongoing infection but bears the unwanted risk of stimulation of potentially harmful autoreactive T cells [24]. Epitope spreading, combined with an increased amount of host hypocretin cell epitopes generated by APCs from destroyed host cell tissue and the adjuvant effect on an infection, may create a fertile field for the development of cerebral autoimmune reactions [25].

In addition to this mode of bystander activation of autoreactive T cells, virus-specific T cells also might initiate bystander activation against hypocretin neurons. For example, virus-specific T cells migrate to areas of virus infection/antigen where they encounter virus-infected cells that present viral peptides in the context of MHC class I molecules to virus-specific T cells [26, 27]. The CD8^+^ T cells recognize these infected cells and release cytotoxic granules resulting in the killing or death of the infected cells. Under these circumstances the dying cells, the CD8^+^ T cells, and inflammatory cells (macrophages) within the inflammatory focus release cytokines such as tumor necrosis factor (TNF), TNF-*β*, lymphotoxin (LT), and nitric oxide (NO), which can lead to bystander killing of the uninfected neighboring cells. This results in additional immunopathology at sites of infection. This also appears to be true for CD4^+^ T cells that can recognize peptide in the context of class II molecules. Cytokines released by the CD4^+^ T cells can directly kill uninfected cells, but also macrophages can kill uninfected cells in a bystander manner [28, 29]. However, this latter scenario seems rather improbable, as MCH neurons are intermingled with hypocretin neurons and MCH-producing cells are intact in narcoleptic patients [13, 14].

## 6. Molecular Mimicry

**Figure 1:**
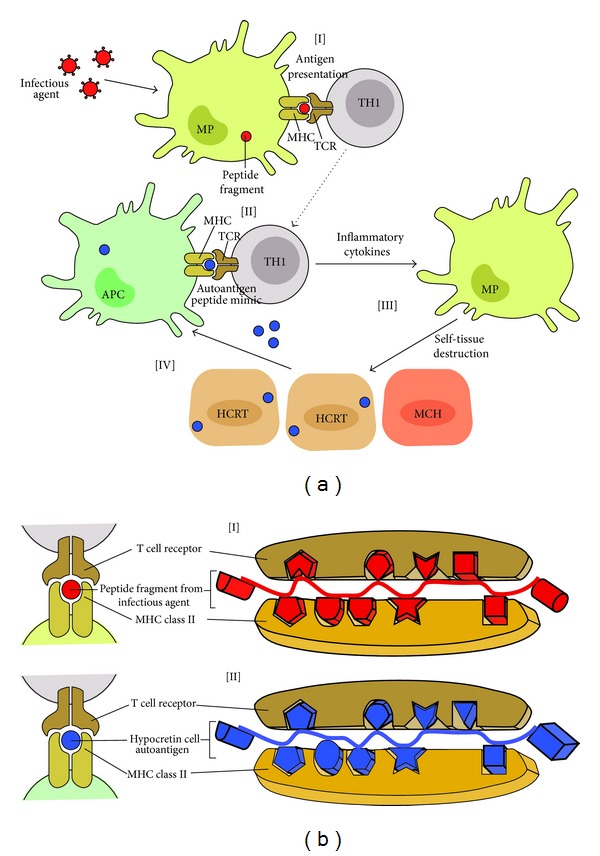
(a) Molecular mimicry describes the activation of cross-reactive TH1 cells that recognize both the microbial epitope [I] and the autoantigen [II]. Activation of the cross-reactive T cells results in the release of cytokines and chemokines [III] that recruit and activate monocytes and macrophages, which mediate self-tissue damage. The subsequent release of self-tissue antigens and their uptake by APCs perpetuates the autoimmune disease [IV]. (b) Molecular mimicry at the MHC/TCR synapses level. Molecular mimicry between infectious agents (H1N1 and/or streptococcus pyogenes) and hypocretin neuron autoantigens. Sequence and structural homology between foreign [I] and self-peptides [II] are required for molecular mimicry to occur. * *The MHC binding groove selects the peptide fragment with a specific amino acid sequence in the context of DQA1∗01:02-DQB1∗06:02. The TCR recognizes a presented peptide with a specific amino acid sequence [I and II].* * Activated CD4^+^ T cells cross-react and recognize hypocretin neuron autoantigens as foreign molecules, prompting an autoimmune response against hypocretin neurons [II].

The attractive hypothesis of the concept of molecular mimicry is based on the existence of structural similarities between antigenic determinants of a pathogen and the host [30] (Figure 1(a)[I]). Consequently, a single T cell receptor may bind to structurally related antigens (Figure 1(a)[I]), which may differ in their amino acid sequence (Figure 1(b)[I] and 1(b)[II]), bound to one or several MHC molecules. This TCR degeneracy implies that T cell responses to pathogen-specific antigens may result in the activation and expansion of T cells (T helper 1, TH1) cross-reactive with self-antigens. TCR recognition is remarkably flexible: a single TCR is able to respond to different peptides (Figure 1(b)[I] and 1(b)[II]) and can react with different peptide-MHC complexes of similar charge distribution and overall shape. Disease-inducing epitopes are those peptides of autoantigens that can be presented by MHC class II molecules on APCs to autoreactive CD4^+^ T cells (Figure 1(a)[II]) [24, 25, 30].

In the case of narcolepsy, molecular mimicry would involve processing and presenting bacterial and/or viral peptides in the context of MHC DQB1∗06:02 (Figure 1(a)[I]), which would activate a population of cross-reactive T cells present in predisposed individuals (Figure 1(a)[II]).

This could easily explain the data obtained following the 2009–2010 H1N1 influenza pandemics, where European investigators reported a significant 6–9-fold increase in the risk of developing narcolepsy after pandemic H1N1 (pH1N1) flu vaccination in Scandinavian children [18–21, 31–37]. It is suspected that vaccination with Pandemrix, a pH1N1 vaccination formulation containing the adjuvant AS03, a combination of squalene and alpha-tocopherol [18, 19], perhaps together with other environmental factors, contributed to the increased incidence of narcolepsy in HLA DQB1∗06:02-positive children. In most, if not all the models where molecular mimicry has been used to induce an autoimmune disease, an adjuvant or an actual infection is required. The fact that the AS03-containing vaccine was particularly associated with increased onset could be the result of the strength and nature of the AS03 adjuvant that would catalyze molecular mimicry between H1N1 proteins and hypocretin cell containing proteins. This suggests that, in addition to having a cross-reacting disease, inducing epitope sufficient activation of APCs is required.

Moreover, the correlation between influenza A infection and the onset of narcolepsy was further strengthened by reports from China showing that onset in children spiked following the H1N1 influenza pandemic of 2009 [21]. Interestingly, the majority (>95%) of these patients had not received H1N1 vaccination, indicating that naturally occurring influenza A infections may increase the susceptibility of developing this disorder.

These findings strongly suggest that some T cells that can be activated by H1N1 epitopes could lead to the destruction of hypocretin-producing neurons.

## 7. Conclusion

The autoimmune basis for narcolepsy is supported by several recent studies from all over Europe, which reported significant increase in the incidence of narcolepsy with cataplexy in children vaccinated with Pandemrix as compared to those in the same age group who were not vaccinated [18–21, 31–37], suggesting that in genetically susceptible children Pandemrix vaccination may alone be sufficient to initiate or precipitate narcolepsy. However, the precise mechanism of disease pathogenesis and etiology still remains an enigma.

Some researchers have hypothesized that narcolepsy could be a B cell autoimmune-mediated disease and that autoantibody production may trigger hypocretin cell destruction. Unfortunately, these studies found moderate [17, 24] to nonexistent production of antibodies [25] in recent-onset narcolepsy patients.

There is evidence for the concept of molecular mimicry playing an important role in this scenario, while bystander activation of T cells appears to be of minor relevance.

It is very conceivable that virus infections, such as influenza A H1N1 virus, could lead to significant activation of APCs such as dendritic cells. These activated APCs could potentially activate preprimed autoreactive T cells, which can then initiate an autoimmune process against hypocretin cells. In addition, virus-specific T cells could migrate to the CNS, where they encounter virus-infected cells that present viral peptides in the context of MHC class I molecules to virus-specific T cells. The CD8^+^ T cells could recognize these infected cells and release cytotoxic granules resulting in the destruction of hypocretin neurons.

Unfortunately, evidence of the involvement of CD8^+^ T cells and MHC class I in the pathology of narcolepsy is virtually nonexistent.

It is rather simple to visualize how molecular mimicry could induce autoimmunity, given that it involves a shared immunologic epitope between a microbe and the host. Disease-inducing epitopes are those peptides of autoantigens that can be presented by MHC class II molecules on APCs to autoreactive CD4^+^ T cells.

The influenza A virus has shown to be one of the most likely environmental factors capable of precipitating autoimmune narcolepsy. Upon influenza A infection, CD4^+^ T cell activation could be achieved after antigen presentation of MHC class II molecules in the context of DQB1∗06:02. It is plausible that these “peptide mimic” specific T cells could cross-react with self-epitopes in hypocretin neurons, thus leading to narcolepsy.

The link between autoimmunity and narcolepsy is clearly a consequence of genetic factors coupled with exposure to environmental factors. The total infectious history of each individual and exposure to other environmental agents has to be considered and tracked. Some of the factors might be disease promoting, whereas others might be protective. In the future it will be important to monitor such environmental factors individually to assess their relative contributions to narcolepsy. The identification of both the underlying autoantigen(s) as well as the causative pathogen is a pending challenge.

## Abbreviations

APC: Antigen-presenting cell
HCRT: Hypocretin
MCH: Melanin-concentrating hormone
MHC: Mayor histocompatibility complex
MP: Macrophage
TCR: T cell receptor.
